# Diagnostic value of mesenteric CTA combined with D-dimer level and inflammatory factor changes in severity of mesenteric artery embolism

**DOI:** 10.12669/pjms.39.5.6719

**Published:** 2023

**Authors:** Yi-ru Zhang, Zhao-yang Li, Jing Liang, Song Bai, Zhu-fen Zhang

**Affiliations:** 1 Yi-ru Zhang, Department of Emergency, Baoding No.1 Central Hospital, Baoding 071000, Hebei, China; 2 Zhao-yang Li, Department of Specialty Nursing, Baoding No.1 Central Hospital, Baoding 071000, Hebei, China; 3 Jing Liang, Department of Specialty Nursing, Baoding No.1 Central Hospital, Baoding 071000, Hebei, China; 4 Song Bai, Department of Emergency, Baoding No.1 Central Hospital, Baoding 071000, Hebei, China; 5 Zhu-fen Zhang, Department of Emergency, Baoding No.1 Central Hospital, Baoding 071000, Hebei, China

**Keywords:** Mesenteric artery embolism, D-dimer, Inflammatory factor, CTA, DSA

## Abstract

**Objective:**

To investigate the value of mesenteric CTA combined with D-dimer (DD) level and inflammatory factor changes in evaluating the severity of mesenteric artery embolism.

**Methods:**

This is a retrospective study. The imaging data of mesenteric CTA and the levels of plasma DD and inflammatory factors in 120 patients with mesenteric artery embolism confirmed by DSA or surgery in Baoding No.1 Central Hospital were analyzed retrospectively from January 2021 to December 2022. The coincidence rate of CTA alone and CTA combined with DD and inflammatory factors with the results of surgery or DSA was compared and analyzed. The specificity, sensitivity and accuracy of CTA alone and CTA combined with DD and inflammatory factors in diagnosing superior mesenteric artery embolism were compared. The correlations of different severity of mesenteric artery embolism with DD and inflammatory factor levels were compared and analyzed.

**Results:**

There was a significant difference in the coincidence rate between CTA diagnosis and CTA combined with DD and inflammatory factors diagnosis (*p=* 0.01). And the sensitivity and accuracy of the latter were significantly higher than those of the former (sensitivity, *p=* 0.01; accuracy, *p=* 0.00). The levels of plasma DD, TNF-a, CRP and IL-6 in the intestinal wall thinning group were significantly higher than those in the thickening group (*p=* 0.00). The above indexes increased significantly in the decreased intestinal wall enhancement group compared with the increased intestinal wall enhancement group (*p=* 0.00). DD, TNF-ɑ, CRP and IL-6 levels increased with the increase in stenosis severity.

**Conclusion:**

Mesenteric CTA combined with plasma DD and inflammatory factor levels can effectively determine the severity of mesenteric arterial embolism, and provide a scientific basis for early clinical diagnosis and treatment.

## INTRODUCTION

Ischemic bowel disease (IBD) is defined as the ischemic damage of the colon and (or) the small intestine due to insufficient blood supply, which can lead to local ischemia and necrosis of the intestinal wall at different degrees, finally resulting in a series of clinical manifestations.^1^,^2^ It is characterized by sudden onset, rapid development, critical illness, difficult early diagnosis, poor prognosis and high mortality.^3^ Therefore, early diagnosis and early severity judgment are of great importance in clinical diagnosis and treatment. At present, the diagnostic methods for this disease are mainly invasive interventions such as surgery and angiography, which have a certain lagging effect. CTA can display thickened arterial wall, calcification shadows on the vascular wall at the lesion site, irregular shape of the vascular lumen and rough vascular wall, but certain specificity is still lacking in the early stage.^4^

In recent years, clinical research data have shown that^5^ intestinal necrosis secondary to mesenteric artery embolism can cause a strong inflammatory response and death. Early intervention can reverse this process and contribute to complete recovery. Plasma D-dimer (DD) is a degradation product of cross-linked fibrin, the production and increase of which reflect the activation of coagulation and fibrinolysis system. In addition, its plasma level can indicate thrombin activity and fibrin production in vivo, and it can be used as one of the indicators for thrombosis in vivo. During the procession of pulmonary embolism, dissecting aneurysm and portal vein thrombosis, the level of plasma DD increases significantly.^6^ On this basis, we used mesenteric CTA combined with DD level and inflammatory factor changes for early diagnosing mesenteric artery embolism and determining its severity, which achieved some results.

## METHODS

This is a retrospective study. The clinical data of 120 patients with mesenteric artery embolism hospitalized in Baoding No.1 Central Hospital were selected from January 2021 to December 2022. Patient data including demographic data, and baseline dosage were retrieved from electronic medical record systems.

### Ethical Approval

The study was approved by the Institutional Ethics Committee of Baoding No.1 Central Hospital(No.:2021-042; May 27^th^, 2021), and written informed consent was obtained from all participants.

### Inclusion criteria:

- Meeting the diagnostic criteria of mesenteric artery embolism;^7^
- Confirmed by digital subtraction angiography (DSA) or surgery;
- Duration of onset < two days without treatment;
- Complete clinical data;
- Clear consciousness without mental illness;
- Willing and able to cooperate on this study, with good treatment compliance;
- Age < 75 years.

### Exclusion criteria:

- Non-initial visit;
- Duration of onset > two days;
- Complicated with chronic inflammation or autoimmune diseases affecting the results. Of the included patients, there were 82 males and 38 females, aging 58-71 years (average, 63.83±7.32 years).
- All patients were visited because of abdominal pain, with a course of disease of two hours-two months.

### CTA scanning

After fasting, the patients firstly received routine plain CT scanning in the supine position using a Philips 64-slice spiral CT scanner. Subsequently, 80 ml iohexol was injected through cubital veins at a rate of three-four ml/s with a high-pressure syringe. Afterward, arterial phase scanning was performed at 24-26 s, portal venous phase scanning at 45-60 s, and delayed phase scanning at 120-180 s. The enhancement of the lesions was observed.

The degree of superior mesenteric artery stenosis was evaluated by two chief physicians observing CTA post-processing images independently. In case of inconsistent diagnosis, the original and reconstructed images were re-observed by the two physicians referring to the international general visual method for evaluation of the vessel diameter.^8^ The calculation formula was as follows: the degree of vascular stenosis = (normal vessel diameter of the proximal part at the stenosis site - vessel diameter at the stenosis site)/normal vessel diameter of the proximal part at the stenosis site × 100%. Superior mesenteric artery stenosis was divided into three degrees: mild, stenosis < 50%; moderate, stenosis = 50%~74%, and severe, stenosis = 75%~100%.^9^ As for indirect signs, the thickness of the small intestinal wall is >3 mm under normal conditions but can be thickened or thinned during ischemia. When the thickness of the colonic wall is > 5mm, it could be diagnosed as intestinal wall thickening. Pneumatosis intestinalis and portal venous gas accumulation are manifested as isolated bubble shadows or banded gas shadows in the intestinal wall or portal vein, which are a diagnostic mark for irreversible intestinal ischemia and of great value in the diagnosis of necrotic bowel.^10^

### Determination methods of DD and inflammatory factors:

*Sample collection:* Before the therapeutic intervention, two ml fasting venous blood was collected from all patients, and the plasma was separated by centrifugation.

*Determination methods:* The content of plasma DD was determined using immunoturbidimetry. The levels of inflammatory factors including tumor necrosis factor-a (TNF-a), C-reactive protein (CRP) and interleukin-6 (IL-6) were detected by enzyme-linked immunosorbent assay (ELISA).

### Observation indexes

1) The coincidence rate of CTA alone and CTA combined with DD and inflammatory factors with the results of surgery or DSA was compared and analyzed. 2) The indirect signs of mesenteric CTA and the levels of DD and inflammatory factors were compared and analyzed. 3) The specificity, sensitivity and accuracy of CTA alone and CTA combined with DD and inflammatory factors in diagnosing superior mesenteric artery embolism were compared and analyzed. 4) The correlations of different severity of mesenteric artery embolism with DD and inflammatory factor levels were compared and analyzed.

### Statistical Analysis

All the data were statistically analyzed using SPSS 20.0. The measurement data were expressed as (*χ̅*± *S*). Data analysis between groups was carried out by the two-group independent sample t-test, and rates were compared using the χ^2^ test. The correlations were expressed as Pearson’s correlation coefficients. *P* < 0.05 was considered statistically significant.

## RESULTS

The comparison of different examinations with surgical or DSA results is shown in Table-I. Among the 120 patients with mesenteric artery embolism confirmed by surgery or DSA, 86 patients were diagnosed by CTA, with a coincidence rate of 71.6%, and 103 patients were diagnosed by CTA combined with DD and inflammatory factors, with a coincidence rate of 85.8%, presenting a significant difference between the two groups (*p=* 0.01).

**Table-I:** Comparison in coincidence rate of different examinations with surgical or DSA results (*χ̅*±*S*) n = 120.

Group	Diagnosis by auxiliary examination	Clinical diagnosis	Coincidence rate*
CTA alone group	86	120	71.6%
Combined group	103	120	85.8%
χ^2^			6.47
p			0.01

* P < 0.05.

The levels of plasma DD, TNF-a, CRP and IL-6 in the intestinal wall thinning group were significantly higher than those in the thickening group (*p=* 0.00). The above indexes showed no significant changes in the increased and decreased intestinal wall density groups (*P* > 0.05), but increased significantly in the decreased intestinal wall enhancement group compared with the increased intestinal wall enhancement group (*p=* 0.00) (Table-II).

**Table-II:** Correlations between CT enhancement alone and its combination with contrast-enhanced ultrasound and pathological results.

CTA signs		n	DD (μg/L)	TNF-ɑ (ng/L)	CRP (mg/L)	IL-6 (ng/L)
Intestinal wall thickness	Thickening	54	1743.86±208.55	34.62±6.53	28.07±7.64	10.83±3.14
	Thinning	48	4762.47±212.83	48.25±9.41	37.40±9.02	16.49±5.11
	t		8.05	8.57	5.64	6.82
	*p* *		0.00	0.00	0.00	0.00
Intestinal wall density	High	33	1893.61±307.82	37.16±7.23	28.46±7.31	12.32±4.16
	Low	56	1876.48±298.96	36.83±7.64	28.13±6.28	13.07±4.32
	t		0.53	0.20	0.23	0.80
	*p*		0.64	0.84	0.82	0.42
Intestinal wall enhancement	Increased	68	1743.40±227.54	32.17±7.59	25.63±7.24	14.02±3.77
	Decreased	71	3407.58±371.40	46.57±8.49	35.81±8.42	18.93±6.08
	t		9.82	8.03	5.79	4.18
	*P* *		0.00	0.00	0.00	0.00

* *p* < 0.05.

Among the 120 patients with mesenteric artery embolism, 86 patients were diagnosed by CTA alone, and 103 patients were diagnosed by CTA combined with DD and inflammatory factors. The sensitivity and accuracy of the latter were significantly higher than those of the former (sensitivity, *p=* 0.01; accuracy, *p=* 0.00), as seen in Table-III. Correlation analysis suggested that DD, TNF-ɑ, CRP and IL-6 levels increased with the increase in stenosis severity, showing positive correlations (Table-IV).

**Table-III:** Diagnostic sensitivity, specificity and accuracy of CT enhancement alone and its combination with PET-CT.

Group	Sensitivity*	Specificity	Accuracy*
CTA alone group	71.6%	88%	73.6%
Combined group	85.8%	93%	95.2%
χ^2^	6.47	1.45	18.00
*P*	0.01	0.23	0.00

* p < 0.05.

**Table-IV:** Correlation analysis of mesenteric artery embolism severity with DD and inflammatory factor levels.

Severity	DD (μg/L)	TNF-ɑ (ng/L)	CRP (mg/L)	IL-6 (ng/L)
Mild stenosis	0.31	0.22	0.28	0.26
Moderate stenosis	0.33	0.27	0.31	0.34
Severe stenosis	0.37	0.30	0.33	0.40

## DISCUSSION

Our study confirmed that among the 120 patients, 86 patients were diagnosed by CTA, with a coincidence rate of 71.6%, and 103 patients were diagnosed by CTA combined with DD and inflammatory factors, with a coincidence rate of 85.8%, presenting a significant difference between the two groups. The levels of plasma DD, TNF-a, CRP and IL-6 in the intestinal wall thinning group were significantly higher than those in the thickening group, with statistically significant differences. The above indexes increased significantly in the decreased intestinal wall enhancement group compared with the increased intestinal wall enhancement group, presenting statistically significant differences. The sensitivity and accuracy of CTA combined with DD and inflammatory factors were significantly higher than those of CTA alone. DD, TNF-ɑ, CRP and IL-6 levels increased with the increase in stenosis severity, showing positive correlations.

Mesenteric artery embolism is a severe acute abdominal disease in clinic, characterized by rapid development and high mortality.^11^ Early diagnosis is the key to the treatment of mesenteric artery embolism.^12^ It has been shown that^13^ the mortality of acute intestinal ischemia caused by mesenteric artery embolism is as high as 50%-90%. For a long time, DSA has been the “gold standard” for the diagnosis of vascular diseases, but it has certain drawbacks and damage. Large-scale DSA for patients with acute abdominal pain has not been recommended by large medical centers.^14^

In recent years, the use of CTA contributes to a more simples, intuitive and accurate diagnosis of vascular diseases, and is easy for patients to accept due to its low cost, rapidness and convenience. CTA can display thickened arterial wall, calcification shadows on the vascular wall at the lesion site, irregular shape of the vascular lumen, rough vascular wall and collateral circulation.^15^ Wiesner et al.^16^ consider that CTA cannot only display the internal conditions of blood vessels (vascular wall and lumen), especially the calcification spots in the lumen, but also accurately show the degree of stenosis of the vascular lumen. The positive predictive value of CTA in diagnosing mild and moderate stenosis of the superior mesenteric artery is significantly lower than its negative predictive value. Schroeder et al. have reported that^17^ the sensitivity and specificity of CTA in the diagnosis of severe superior mesenteric artery stenosis are both significantly higher than those in patients with mild stenosis, which may be related to the improvement in time and density resolution of dual-source CT compared with previous multi-slice spiral CT.

In patients with mesenteric vascular embolism, the elevation in DD and inflammatory factor levels indicates acute intestinal ischemia. Moreover, Acosta et al.^18^ used these indexes as a sign of emergency laparotomy. Plasma DD is a degradation product of cross-linked fibrin. Its production or increase reflects the activation of the coagulation and fibrinolysis system. Its plasma level can indicate enzyme activity and fibrin production in vivo and can be used as one of the indicators for thrombosis in vivo. Khripun et al.^19^ believe that DD detection can diagnose mesenteric artery thrombosis earlier and shorten the preoperative time. Additionally, CRP, IL-6 and DD are identified as biomarkers for the diagnosis of mesenteric ischemia. Onody et al.^20^ also confirmed that after ischemia-reperfusion injury, the levels of inflammatory factors such as IL-6 and TNF-a increased significantly, and anti-inflammatory treatment could not only reduce the intensity of local injury, but also decrease the intensity of the systemic injury.

### Limitations

The limitations of this study lie in the small sample size and the lack of follow-up. The study content was not combined with the final outcomes of the patients. In future clinical work, we will continue to enlarge the sample size and supplement follow-up. In addition, mesenteric CTA, DD level and inflammatory factor changes will be combined with the outcomes of patients, so as to more objectively and comprehensively describe the advantages and disadvantages of this examination method, and benefit more patients.

## CONCLUSION

Mesenteric CTA combined with plasma DD and inflammatory factor levels can effectively determine the severity of mesenteric arterial embolism and provide a scientific basis for early clinical diagnosis and treatment. Therefore, it has a certain clinical value.
